# Shaping in the Third Direction; Synthesis of Patterned Colloidal Crystals by Polyester Fabric-Guided Self-Assembly

**DOI:** 10.3390/polym13234081

**Published:** 2021-11-24

**Authors:** Ion Sandu, Claudiu Teodor Fleaca, Florian Dumitrache, Bogdan Alexandru Sava, Iuliana Urzica, Iulia Antohe, Simona Brajnicov, Marius Dumitru

**Affiliations:** Lasers Department of National Institute for Lasers, Plasma and Radiation Physics, 409 Atomistilor Street, 077125 Bucharest, Romania; ion.sandu@inflpr.ro (I.S.); claudiu.fleaca@inflpr.ro (C.T.F.); florian.dumitrache@inflpr.ro (F.D.); iuliana.iordache@inflpr.ro (I.U.); iulia.antohe@inflpr.ro (I.A.); simona.brajnicov@inflpr.ro (S.B.)

**Keywords:** colloidal crystals, polyester fabric, polystyrene, silica, sub-micron spheres, self-assembly, negative diffraction, super-prism effect

## Abstract

A polyester fabric with rectangular openings was used as a sacrificial template for the guiding of a sub-micron sphere (polystyrene (PS) and silica) aqueous colloid self-assembly process during evaporation as a patterned colloidal crystal (PCC). This simple process is also a robust one, being less sensitive to external parameters (ambient pressure, temperature, humidity, vibrations). The most interesting feature of the concave-shape-pattern unit cell (350 μm × 400 μm × 3 μm) of this crystal is the presence of triangular prisms at its border, each prism having a one-dimensional sphere array at its top edge. The high-quality ordered single layer found inside of each unit cell presents the super-prism effect and left-handed behavior. Wider yet elongated deposits with ordered walls and disordered top surfaces were formed under the fabric knots. Rectangular patterning was obtained even for 20 μm PS spheres. Polyester fabrics with other opening geometries and sizes (~300–1000 μm) or with higher fiber elasticity also allowed the formation of similar PCCs, some having curved prismatic walls. A higher colloid concentration (10–20%) induces the formation of thicker walls with fiber-negative replica morphology. Additionally, thick-wall PCCs (~100 μm) with semi-cylindrical morphology were obtained using SiO_2_ sub-microspheres and a wavy fabric. The colloidal pattern was used as a lithographic mask for natural lithography and as a template for the synthesis of triangular-prism-shaped inverted opals.

## 1. Introduction

Colloidal solutions containing spheres of sub-micron dimensions can form through the phenomenon of self-assembly following the loss of their liquid by evaporation, an ordered porous solid known as colloidal crystal (CC) [1,2,3,4]. As one-dimensional (1D) or bi-dimensional (2D) materials, they are challenging, by virtue of their low cost, to sophisticated but expensive technologies, such as photolithography [5], direct laser writing [6], two-photon polymerization [7], and holographic methods [8], when competing for the production of photonic crystals (PCs) [9,10,11]. There are two winning strategies in this competition. In the first, CCs are used as templates by filling their interstitial spaces with a fluid precursor capable of solidification. Then, the templates are removed to obtain a porous inverse replica [12]. In the second strategy, CCs are used as masks in colloidal lithography [13], where a mono or bilayer of colloidal crystals is exposed to reactive ion etching to produce surfaces with heterogeneous chemistry or to metal evaporation to produce arrays with nanometric features over a large area. In both cases, the resulting structures can be defined as PCs, namely periodic dielectric structures on the light wavelength scale, which can manipulate light in the same manner as a crystal lattice manipulates electrons [9]. As in the case of PCs, their most important property is Bragg diffraction of light [14]. Thus, they present optical band gaps (range of frequencies and directions through which light cannot pass) [15]. As is the case with PCs, CCs can be used as opto-chemical and opto-biological sensors [16,17] to detect chemical and living events, as band-stop and band-pass filters [18,19] for integrated optics, as micro-antennas [20] for satellite and mobile communications, or as laser components [21].

As three-dimensional (3D) materials, CCs compete with three-dimensionally-ordered macro-porous (3DOM) materials obtained by means other than self-assembly methods [22]. In this case, voids in the volume of CCs and the interconnectivity of their voids are useful in phenomena involving the movement of fluids through pores. Such movement of fluids is not facilitated by CCs themselves but by their porous inverse replica, which results when a 3D CC is filled with a fluid precursor capable of solidification, followed by removal of the template. As 3DOM materials, they can serve as catalysts [23,24], porous electrodes [25], or membranes for smart filtration [26], among other applications. Even if they look similar to microporous and mesoporous ordered materials (such as zeolites [27] and metal organic frameworks (MOFs) [28]), the main difference consists in their pore size. Whether CCs are produced as PCs or 3DOM materials, they must present a high level of ordering both for Bragg diffraction to exists and for a decrease in the tortuosity of materials (diffusion and fluid-flow easiness in porous media) [29].

Usually, simple techniques, such as sedimentation, LB technique, floating packing, spin coating, drop drying, vertical deposition, and others [30], produce colloidal crystals with flat extended surfaces flat parallel with the substrate, having sub-micron spheres arranged only in a hexagonal or cubic packing, which repeats in all three directions. Expensive and sophisticated technologies [5,6,7,8] can synthesize almost any highly ordered, reproducible array or pattern that can be designed and fabricated as thin film. However, both approaches fail in the third-direction periodical structuring of PCs. Simple techniques can present only hexagonal or cubic structuring, and expensive techniques can repeat the structuring of the extending surfaces only along a few layers. Optical transmission properties of PCs and CCs as PCs depend on the number of structured layers (between one and dozens) in the third direction [31]. In the great majority of cases, self-assembled colloidal crystals exhibit only (111) dense-packed planes at the surface [32] or in some cases, on the fcc (100) or bcc (100) [33], (001), or (110) planes [34]. The simultaneous controlled presence of multiple types of surface planes in these photonic crystals would greatly enrich their photonic bands and, consequently, their potential applications. A curved surface exposes different packed planes, and this can be seen in round colloidal crystals, such as spheres, hemispheres, cylinders and rings [35,36,37,38]. Compared with the flat surfaces of CCs, which present different colors when observed from different angles, the curved surfaces show the same color, which is independent of the viewing angle [39,40]. Unfortunately, other interestingly shaped colloidal crystals, such as flat surfaces that are not parallel with the substrate surfaces, like pyramids or triangular prisms, or curved but concave (as opposed to convex) surfaces, as in the case of spheres, hemispheres, cylinders, or rings, were rarely obtained by self-assembly. However, there are a few approaches through which we can increase the physical or chemical properties of an ordered, porous thin film. The first is to fabricate thin crystals onto a curved substrate [41]. The second is, as mentioned before, to shape the film as a three-dimensional object. The third approach is to produce hierarchical order by patterning a substrate with small crystal units. These patterns have minimized volumes and integrated functions, compared to traditional film devices, and provide better performance than their structural units [32]. Usually, the patterns are designed by using the same techniques employed for obtaining CCs units; thus, the absence of complex structuring of CC units in the third dimension persists.

There are two approaches that are usually used for the fabrication of patterned colloidal crystals (PCCs). In the first, sub-micron spheres self-assemble as a film, which are patterned using plasma etching [42], ultra-sonication [43], lift-up soft lithography [44], and other methods. In the second, the substrate is patterned first, and on this patterned substrate, the sub-micron spheres self-assemble in the proper zones. These zones are restricted from the rest of the substrate by chemical wettability [45,46], physical confinement [47,48], the most used method of which is ink-jet printing [49], and surface relief [50,51]. However, in almost all cases, the resulting patterns are rather poor, limited to simple bi-dimensional arrays of flat lines and dots. Moreover, by using expensive technologies for substrate pre-patterning, the overall cost greatly increases. In the first approach, it is impossible to sculpture sub-micron sphere film other than normally to the surface. In the second approach, even when sub-micron sphere film can be precisely cut or etched in the lateral extension, it is limited to the spherical [52] or flat liquid/air interface shape of the self-assembly zones. This induces a spherical or flat shape of the final colloidal crystals, leading to the poor structuration of colloidal crystals in all three dimensions. A pyramid, for example, is much more difficult to achieve, especially trough a pure self-assembly phenomenon. However, self-assembly of sub-micron spheres on a surface-modified relief seems to offer some possibilities. If “positive-relief” rectangle-shaped colloidal crystals could be produced [50], pyramid-shaped crystals could only be obtained only by using a “negative” relief [53,54]. In both cases, the most important problem is that after self-assembly, the colloidal crystals remain trapped in their relief. Their most important surfaces— those which are not parallel with the substrate—remain closed to the external medium.

However, assuming that we can fabricate a single-micrometer prism by self-assembly—meaning to succeed in shaping the capillary forces involved in the process—its multiplying and ordering in a pattern requires repetition on a large scale of the local conditions necessary for the self-assembly of a single prism. This means that the capillary forces that act on the micrometer scale must be able to repeat in a pattern on the macroscopic scale. We found that a commonplace material, more precisely a polyester microfiber woven fabric [55], can fulfill these conditions. The fabric can be seen as a lithographic mask, but comparing with it a fabric presents some remarkable properties regarding its use in the patterning of substrates. It has knots [56]. These knots allow the fibers of the fabrics to settle towards the substrate at a certain distance. Thus, the colloidal liquid can wet the substrate below each fiber. A fabric in close contact with a smooth solid substrate will form a kind of three-dimensional lithographic mask. A colloidal solution deposited between substrate and fabric will allow the movement of the liquid-dispersed nanometer or micron solid particles over a much longer distance than a single unit, as in the case of a lithographic mask. Moreover, between the hydrophobic polyester fibers and the hydrophilic substrate, a special air/liquid interface will form in the zone of each opening, which will act not only to induce their own shape to the final solid pattern, but these interfaces will strongly confine the sub-micron spheres during solvent losses, providing the necessary force for the self-assembly of a quality colloidal crystal. However, the most important thing is that after sub-micron spheres self-assemble in the dry-patterned colloidal crystal, the fabric can be completely peeled off. It is worth mentioning that a specific research domain in materials physics, namely “capillary-bridge-mediated-assembly” or “liquid-bridge-induced assembly (LBIA)” [57,58,59], may offer some suggestions on the synthesis of patterned and shaped colloidal crystals. Even though their main purpose is to obtain 1D nanostructures to be used as waveguides and they usually use nanometric particles and not sub-micron spheres, the fact that they are based on capillary forces that confine nanoparticles through liquid bridges built into a different architecture than the methods cited in reference [30] could help us. Unfortunately, this approach also uses expensive technologies.

In this work, we present, for the first time, a cheap and fast method by which a new kind of patterned colloidal crystal can be fabricated by using a hydrophilic substrate, a water-based sub-micron-sphere colloidal solution, and a hydrophobic polyester fabric. A sub-micron sphere can thus self-assemble with remarkable three-dimensional ordered concavities, much similar to those possessed by some living creatures [60].

## 2. Materials and Methods

### 2.1. Materials

Polystyrene (PS) sub-micron-sphere aqueous colloidal solutions with 0.150 µm, 0.300 µm, 0.488 µm, and 20.0 µm and SiO_2_ with 0.245 µm mean diameter, 5% *w*/*v*, were purchased from microParticles GmbH, Berlin, Germany, and used as they were or diluted with deionized water when needed. Commercially available microscope glass slides, optical polished silicon, and steel samples were used as substrates after a few minutes cleaning by ultrasonication in acetone, distilled water, and ethanol, followed by natural drying. Commercially available polyester fabric sheets (polyester veil) were cut in 1 cm × 1 cm pieces and used as they were. However, most appropriate materials can be found on the Internet by using the keywords: polyester, plain, precision, fabric.

### 2.2. Synthesis of Patterned Colloidal Crystals

**Figure 1:**
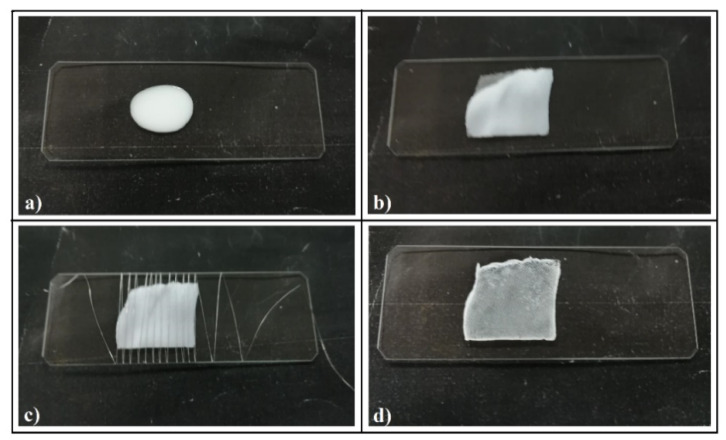
Experimental set-up and the necessary steps for synthesis of colloidal crystal self-assembly guided by polyester fabric: (**a**) colloidal solution drop on the microscope glass slide substrate; (**b**) polyester fabric pressing the colloidal drop; (**c**) metallic wire rolled around the fabric and the colloidal film; (**d**) the final dried PCCs after the wire and the fabric were removed.

A few droplets of sub-micron-sphere colloidal solution were deposited onto the surface of a microscope glass slide (Figure 1a).

A piece of fabric sheet of ~1 cm^2^ was gently deposited onto the colloidal drop and lightly pressed to stick it to the glass substrate (Figure 1b). A thin metallic wire (100 μm diameter) was rolled tight around the fabric and colloid (the distance between metallic spires was 2–4 mm) (Figure 1c). The role of the metal wire is to keep the fabric in tight contact with the substrate throughout the evaporation of the solvent and, at the same time, to introduce as little disturbance as possible to the natural evaporation process. Several types of wires were tried—metallic, polymeric (from polyester or cellulose)—but the copper wire was proven to best meet these conditions. Close contact between the fabric and the substrate throughout the evaporation of water is necessary because the fabric, if left free, undergoes some square-millimeter detachments from the substrate in the final phase of evaporation (inhomogeneous evaporation). After a few minutes (~30 min in the normal conditions of temperature and humidity of the laboratory, T = 25 °C, relative humidity (RH) = 40–60%), once the liquid completely evaporated, the wire and fabric were removed and the colloidal crystal can be further used in experiments (Figure 1d). We mention that the same result was obtained if we first placed the fabric on the empty substrate, rolled the metal wire and then deposited a few drops of colloidal solution on top, slightly tapping it.

Thin-film colloidal crystals of 488 nm PS spheres were also fabricated by the spin-coating technique using a WS-400BX-6NPP/LITE model (Laurell Technologies, New Wales, PA, USA). A few drops of colloidal solution (PS 488 nm, 5% *w*/*v*) were poured onto the microscope glass slides used as substrates, which were spun at 1500 rpm for 30 s.

### 2.3. Investigation

Macro-scale observations of the as-synthesized patterned colloidal crystals were performed by using optical microscopy and a digital photo camera. A scanning electron microscope (SEM) (Apreo S Thermo Fisher Scientific, Auburn, AL, USA) and an atomic force microscope (AFM) XE-100 (Park Systems Inc., Santa Clara, CA, USA) were used to observe the structures and morphologies of the self-assembled patterned colloidal crystals at sub-micron and nanometric scales. A thin layer of gold was sputtered onto the samples prior to imaging. UV—vis transmittance spectra were acquired by using an optical-fiber-connected AvaLight-DHc light source (spot size ~200 μm) and an AvaSpec-ULS2048CL-EVO—high-resolution spectrometer, all from Avantes, Apeldoorn, The Netherlands.

## 3. Results and Discussion

**Figure 2:**
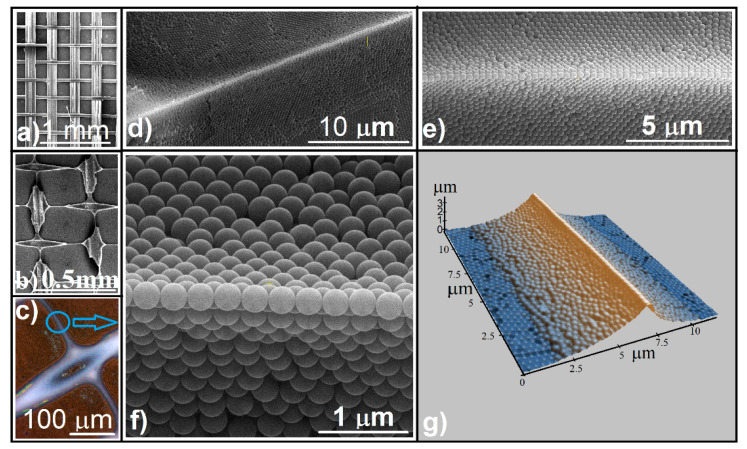
Patterned and shaped colloidal crystal: (**a**) SEM image of the fabric structure; (**b**) SEM image of the patterned crystal; (**c**) optical microscopy image of a cells-unit intersection; (**d**–**f**) SEM images of triangular prisms of colloidal crystals; (**g**) AFM image of a triangular prism.

By looking through a magnifying lens, we were able to observe a regular grid (Figure 1d), which seems to reproduce the fabric design (Figure 2a). SEM imaging (Figure 2b) shows regular square cells of around 0.35 mm in size with a small, round opening (a few dozen micrometers) in the center of each cell. Sparkling, solid walls and a pale yellow or brown surface between walls and the centered opening was observed by reflection optical microscopy (Figure 2c). Upon closer examination (SEM), magnificent triangular prisms comprising well-ordered sub-micron spheres in a hexagonal array stay straight for hundreds of micrometers in length (Figure 2d–f), each prism finishing on its top with one dimensional array of sub-micron spheres (Figure 2f). AFM investigations show that the prism surfaces are slightly concave, and the width and height of prisms are around 10 µm and 3 µm, respectively (Figure 2g).

The ordering and the reproducibility of these structures are remarkable, obtained every time the experiments were performed under the same conditions. However, during a large number of experiments, we noticed the following general trends:

(a) Colloidal concentrations greater than 1% are needed for prisms to appear, and concentrations greater than 3% are needed for a line of spheres to settle on their upper edge. These values should not be taken as absolute but rather as starting values. They can vary within certain limits with all the parameters upon which self-assembly of the spheres depends.
(b) The ordering quality increases with the increase in the hydrophilicity of the substrate. Although prisms can be obtained even on polymeric substrates, the best results are obtained on glass substrates.
(c) The overall quality of the grids slightly increases as the size of the spheres decreases.
(d) The shape of the unit cell and the CC pattern are imposed by the shape of the fabric cell and its pattern, at least within the limits of our experiments (polyester fiber diameter of tens of micrometers, fabric openings between 300 and 1000 μm).
(e) The quality of the obtained grids depends on the hydrophilicity of the fabric. Less hydrophilic fabrics obtain better results.
(f) The self-assembly phenomenon using fabric is less sensitive to external parameters, such as ambient pressure, temperature, humidity, or minor vibrations or shocks.
(g) The structure of the grids and the ordering quality of the sub-micron spheres do not change with the variation in a wide band of liquid volatility. Experiments performed in which the liquid-medium volatility (evaporation rate) was extremely low (RH ~99%) or very fast (evaporation inside the oven, T = 30–80 °C, RH < 10%) did not show changes in the ordering quality of the sub-micron spheres, although the evaporation time of the same volume of colloidal solution varied between several days and a few minutes.

**Figure 3:**
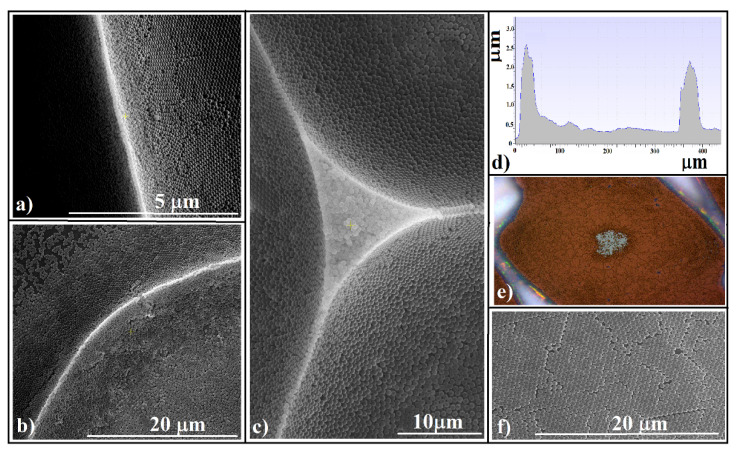
SEM images of: (**a**) Straight triangular prism; (**b**) curved triangular prism; (**c**) knot; (**d**) profile of a unit cell; (**e**) reflection optical microscopy of a unit cell; (**f**) SEM image of PS single layer from the inside of unit cell.

The unit cells could be described by means of three basic components:

(a) Triangular prisms (Figure 3a), which can be straight or curved in 2D, depending on the shape of the fabric opening.
(b) Knots (Figure 3c), whose shape also depends on the structure of the fabric and the number and thickness of fibers that intersect at each node of the fabric. Although spheres are tightly packed in the volume of these structures, the surface layers are often disordered. The cause could be the “late evaporation” [61,62] of the solvent, but as long as ordered surfaces are sometimes obtained, there is the possibility that this phenomenon can be controlled.
(c) The interior of the unit cells has an almost flat bottom (Figure 3d) and consists mainly of a monolayer of tightly packed spheres with an empty space of a few tens of micrometers, in the center (Figure 3e). The degree of ordering is good enough (Figure 3f) that the spheres form a polycrystalline structure, each mono-crystalline domain having several tens of micrometers in size, large and packed enough to produce the Bragg light diffraction phenomenon. However, bilayers zones, amorphous regions, and packing defects are often found. Remarkably, no crack was observed at any level.
(d) The quantification of the order quality was done through the parameter “range order, RO”, defined by us and published in a previous paper as “the result of multiplication between the mean area of a perfect domain and the number of nanospheres that it contains” [63]. By analyzing the SEM images of our patterned colloidal crystals, we measured some values of the range-order parameter (Table 1).

**Figure 4:**
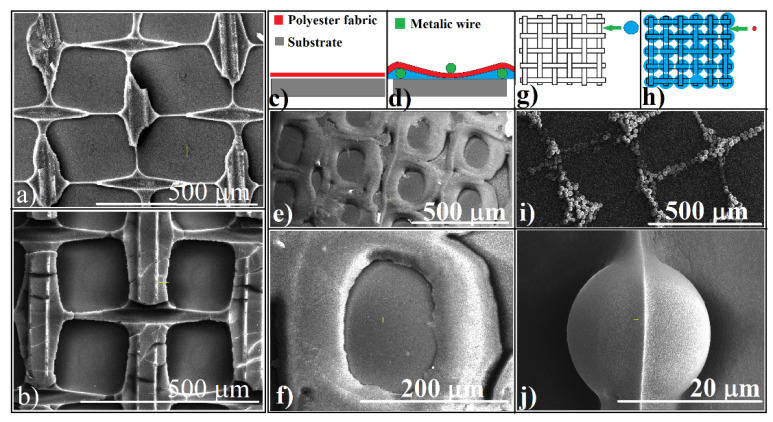
(**a**) SEM image of a colloidal crystal grid where 5% PS sphere concentration was used; (**b**) SEM image of a colloidal crystal grid where 20% PS sphere concentration was used; (**c**) schematic of fabric onto the substrate in a normal configuration; (**d**) schematic of wavy fabric onto the substrate; (**e**,**f**) SEM images of the grid and unit cell resulted by using silica spheres and wavy fabric; (**g**,**h**) schematics of the stepped process for colloidal-crystal self-assembly guided by polyester fabric; (**i**) 20 μm PS spheres forming a colloidal crystal grid; (**j**) 0.150 μm PS spheres covering layer onto 20 μm PS spheres.

These were the three structural components we encountered each time, no matter what fabric, spheres, solvent, or substrate we used. Thus, we have colloidal crystals shaped as triangular prisms, somehow curved truncated pyramids (knots), and cavities, which present large areas of slightly concave surface. However, an increase in shaping complexity could be achieved by performing some specific experiments. The simplest one is to use colloidal solutions with high concentration. If by using colloidal solutions with concentrations of 3–5%, the structures mentioned above are obtained (shown for comparison in Figure 4a), then by using concentrations higher than 10–20%, the solid structures self-organized using fabric and change their shape. Although they retain the organization imposed by the fabric, the solid walls are no longer prismatic but take on the shape of the fibers that make up the fabric (Figure 4b), a kind of negative shape of the fibers. The fabric film becomes a mold. The monolayers inside each cell become multilayered, and the central area, empty of spheres, is completely covered.

Unfortunately, the degree of ordering decreases sharply, and future experiments would be needed to find the conditions under which we can keep both the ordering of the spheres and the shapes induced by the fibers.

A second approach by which we can change the shape of self-organized structures is to unfold the fabric itself into a new dimension. The normal fabric (50 μm in thickness) that is usually stuck to the substrate (schematic in Figure 4c) has been wavy by rolling a parallel metallic wire of 100 μm in diameter onto the substrate, followed by a second wire, which was rolled onto the sample after the colloid and fabric were deposited (Figure 4d). The distance between two consecutive parallel metallic wires was of 2 mm, whether they are above or below the fabric. Thus, a variable distance between fabric and the substrate is obtained. In this case, the self-assembled structure keeps the grid pattern (Figure 4e), but the prisms no longer form. Instead, semi-cylindrical walls border each unit. The wall diameter reaches 100 μm (Figure 4f), and their surfaces present a high quality of nanosphere ordering.

A third viable strategy by which we can change the shape of self-organized structures is to take advantage of a method characteristic, namely that multiple deposition can be performed in a sequential manner as long as the previous structure is not dislocated or dissolved. Thus, multi-layered structures can be achieved. As an example, if we first deposit a 20 μm microsphere suspension, although it is at the maximum limit of the size of the spheres that can still be organized (Figure 4g), after evaporation of the solvent, a quantity of colloidal solution containing much smaller spheres can be applied—in our case, 0.15 μm PS spheres (Figure 4h)—which begin to conformally deposit over the prisms composed of large spheres (Figure 4i), forming a coating with a ridge on their surface with 1D spheres on its upper edge (Figure 4j).

We present three possible applications of patterned colloidal crystals formed by self-assembly and guided by a fabric, although the actual number of applications is probably much higher.

**Figure 5:**
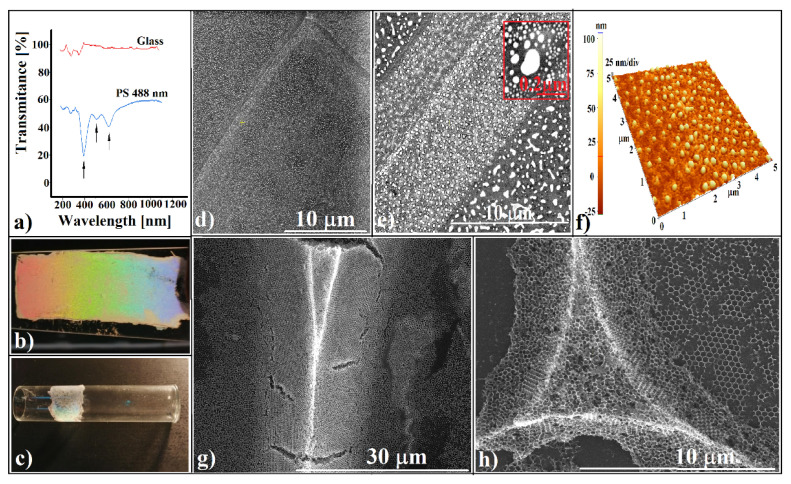
(**a**) UV-Vis spectra of white light transmission trough a patterned, shaped colloidal crystal; (**b**) image of super-prism effect of a patterned and shaped colloidal crystal formed onto a flat substrate; (**c**) image of super-prism effect of a patterned and shaped colloidal crystal formed onto a curved substrate; (**d**,**e**) SEM images of the Au nanometric array resulted by using a triangular prism colloidal crystal as lithographic mask; (**f**) AFM image of the Au nanometric array; (**g**,**h**) SEM images of a triangular prism and a knot, inverted opals.

As we mentioned before, Bragg diffraction of light is a common phenomenon in colloidal crystals. Usually, the quality of such colloidal crystals is given by the height and narrowness of the reflection bands or by the depth and narrowness of the transmission bands. The number of features and their position are imposed, on the one hand, on the sphere’s nature and size, and on the other hand, on the sphere’s packing. The most important part of the self-assembly method forms a hexagonal close-packed (hcp) monolayer, and thicker film can be seen as stacks of this single-layer. There are three ways of stacking the layers: hexagonal, cubic-centered face (cfc), and double hexagonal [64]. Each method presents distinct features in reflected or transmitted light. UV-vis. spectrometry measurements performed on our samples show the existence of three dips in transmitted light (Figure 5a).

The dips are placed at: λ_EXP_ = 620 nm; 510 nm; and 390 nm. First, we must remark on the absence of the most important dip corresponding to 3D CCs with a cfc packing. The Bragg light diffraction on its (111) planes [65] it should have generated a minimum placed at 1172 nm, and in the case of polystyrene spheres, 488 nm. Second, the transmission bands from Figure 5a fit very well with the band gap’s positions found by other authors [66,67] when they studied Bragg light diffraction on a polystyrene hexagonal close-packed sphere as a monolayer. They showed that dips in the transmission spectra arise at the spectral positions where parameter $Z=\frac{\sqrt{3}d}{2\lambda }$ satisfies condition *Z* = 0.71; 0.85; 1.00; 1.34, or 1.55 [66], where *λ* is the dip position and d is the sphere diameter. Verifying our wavelength positions for a 488 nm polystyrene sphere, we found, for *Z,* the next values: *Z* = 0.68; 0.85; 1.08. All of these, as well as the profilometry image from Figure 5d, suggest that inside of each unit cell of the patterned CCs, a single layer of sub-micron spheres is formed. The difference between the theoretical values and experimental values can be attributed to the stacking faults and to the mixture of single layers with double layers in some proportion. Because an identical spectrum was obtained on the samples produced by spin coating where no prism was present (not seen at SEM images), we can conclude that the role of triangular prisms is absent or minor in Bragg diffraction of light on our patterned CCs.

An important property of CCs is their negative refractive index, which induces left-handed behavior of the diffracted light [68]. Because accurate measurements of this phenomenon can be performed only through a special optical experimental setup, in its absence, we resorted to simple photos, which can be seen in Figure 5b (the white light dispersion when, it passes through a patterned CC, forms 488 nm PS spheres onto a flat substrate on microscope glass slide). We also used rough geometrical measurements (angles at which a spot from the sample changes its color from dark blue to light red). The incident angle was higher than 20°. Thus, we measured an angular dispersion of around 6 nm/degree. However, the samples synthesized by spin coating showed the same phenomenon and the same angular dispersion value; thus, with the super-prism effect, we cannot assign an influence of the triangular prisms. Even so, accurate measurements and/or an intelligent designed experimental setup might evidence their possible properties of light manipulation, such as waveguides, for example.

The super-prism effect could also be seen in our patterned colloidal crystals formed onto a curved substrate (glass cylinder) (Figure 5c). The self-assembly of sub-micron spheres onto curved substrates is a great challenge [69]. However, we found that this is possible if the fabric is highly elastic (a sheet of women’s stocking, for example). We found that even by using a normal polyester fabric sheet rolled onto a curved substrate, grids and prisms formed; the sphere single layers became completely disordered but kept their ordering if the fabric sheet makes a tight contact with the curved substrate.

A second application of the sub-micron sphere grid that generates some interesting results is its use as a lithographic mask. As mentioned in the introduction, arrays of nanometric structures can be obtained by using the interstice between the solid spheres by chemical etching or by depositing various materials from the vapor phase. A close-packed colloidal assembly usually forms an array of triangular-shaped structures below the single layer or a hexagonal array of a quasi-hexagonal nanodots in the double-layer case [13]. We present, for the first time, what results if a 3D prismatic opal is used as template for colloidal lithography. In fact, the sample used for close viewing under SEM (Figure 5d,e), the surface on which a thin gold film was deposited by sputtering, was calcinated for PS sub-micron-sphere removal and reanalyzed by SEM. Somewhat surprisingly, after calcination, a shaded material structure could be seen in the SEM images (Figure 5d), which seems to reproduce the triangular prisms from Figure 3. We assume that this shadow is induced by the difference in dot surface density generated by the angle at which the gold source irradiates the sample (60° relative to the substrate) [70]. If the irradiation angle is different from 90°, the Au atoms will first hit the nearest colloidal prism surface, depositing a higher Au density than in the zone of the opposite surface. Upon closer inspection, we can see a hexagonal array of slightly ellipsoidal gold dots (non-circular shape could be also an argument of angle deposition effect), each one of them surrounded by a few smaller satellites, and so on, in a fractal-like organization (Figure 5e inset). By using PS sub-micron spheres of 488 nm in diameter (14 nm standard deviation) as a mask, the mean sizes of Au dots measured from SEM images (100 measurements) were of 126 nm for the first-order dots (26 nm standard deviation) and 50 nm (10 nm standard deviation) for the second-order dots (satellites). AFM investigations (Figure 5f) reveal a height of around 50 nm of the first-order gold dots. The intriguing question (for which we have no answer) is how the gold vapors penetrated so many sub-micron-sphere layers (more than six) to fix and form Au dots at the bottom of and in the central part of the prisms.

The third application of our colloidal crystal is using it as a template for the synthesis of inverted opal structures. By infiltrating a 25 wt.% Na_2_S_i_O_3_ water-based solution in the patterned colloidal crystal obtained by using a fabric and drying and calcinating it at 400 °C, we obtained an inverted replica of the initial patterned colloidal crystal (Figure 5g,h). However, the most important finding was to obtain a skin-free structure during solution infiltration because helping techniques such as microtome cutting or etching processes do not work to remove the overlayer deposited onto the colloid prism’s surface. The quality of our inverted opals must be further improved (we are still working at this), and interesting applications of such shaped crystals must be imagined. The most important quality of a triangular-prism-shaped material is its capacity to disperse an incident-coherent beam in many more secondary, angular-dependent, refracted beams when the beam interacts with the non-parallel surfaces of the prism. For non-optical phenomena, such as atoms, molecules, or nano-objects flowing through the prism, the different distances that they cross between the two non-parallel walls can create inhomogeneities, anisotropies, or useful gradients after their exit from the prism. The triangular shape of colloidal prisms can be used for improving some parameters of applications that are already used for the “microprism array” key concept. Thus, they might be used for directional transmission of light [71] or for improving the efficiency of solar cells [72]. Interesting fields of application might be opto-fluidics [73] and micro-fluidics [74] if the triangular prisms are fabricated as inverted opals, as wave guiding 1D structures [75], if they could be fabricated in the nanometric domain or as cell and tissue scaffolds [76], or if they could be fabricated in the millimetric domain.

However, this work is a proof of concept. We prefer to suggest a number of interesting future applications rather than to systematically investigate a single one of them. We consider it a huge challenge to correlate the fabric architecture with experimental setup parameters and final properties of the patterned colloidal crystals and to predict the colloidal-crystal unit shape and the packing structure of the sub-micron spheres in each unit. The third dimension of colloidal crystals must be conquest!

**Figure 6:**
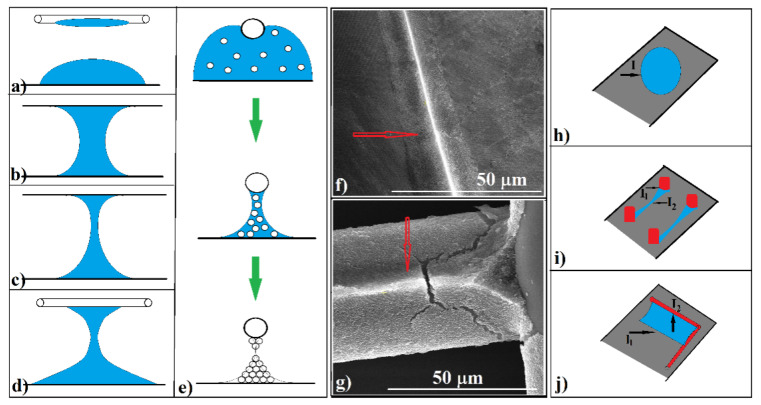
(**a**) Schematic of water droplet resting on a flat substrate or hanging on a fiber; (**b**) schematic of a liquid bridge formed between two parallel, flat, similar substrates; (**c**) schematic of a liquid bridge formed between two parallel, flat, dissimilar substrates; (**d**) schematic of the possible shape of a liquid bridge formed between a flat substrate and a fiber; (**e**) liquid bridge shape changing during evaporation; (**f**) SEM image of the colloidal crystal shaped as a triangular prism onto the flat substrate; (**g**) SEM image of the colloidal crystal shaped as a triangular prism onto the curved fiber surface; (**h**) schematic of solid/liquid/gas interface, which appears in the usual colloidal-crystal self-assembly methods; (**i**) schematic of solid/liquid/gas interfaces, which appear in the “capillary-bridge-mediated-assembly” technique; (**j**) schematic of solid/liquid/gas interfaces, which appear in the “fabric-guided self-assembly” technique.

At the end of this paper, we will try to briefly show how these triangular prisms may form. The mathematics and physics behind this apparently simple phenomenon are of a high complexity, many of the specific equations requiring numerical simulations. Therefore, first of all, we consider that fabric-guided self-assembly takes place on modified-relief patterned surfaces. On such surfaces, the capillary forces, which act for sub-micron spheres confinement, are the result of interplay between the hydrophobicity of substrate and fabric fibers. The adhesion force between water and fibers or substrate is much higher than the cohesion force between water molecules. During solvent evaporation, water from the center of each unit cell will be moved closer to each fiber, thus leaving an empty space in the center of each cell. When only a small part of the initial colloid solution remains in the system, the liquid bridges that form between the substrate and fiber surfaces impose the shape of the final solid deposits. A water droplet that rests on a flat substrate and one that hangs on a fiber can take (in conditions close to ours) the shape shown in Figure 6a. A droplet that simultaneously wets two close, flat, parallel, and chemically similar substrates takes the shape shown in Figure 6b. If the substrates present dissimilar wetting properties, the liquid-bridge shape changes, as in Figure 6c. Thus, the shape of the colloid liquid bridge between the substrate and the polymer fibers of the fabric could look like Figure 6d. In this case, the dynamics of the liquid bridge shape during evaporation could be close to those in Figure 6e. The capillary forces that appear at the interface between the liquid and the two solid bodies, polyester fiber and glass substrate, which have different wettability properties, have different orientations and sizes so that their result generates a volume of liquid in the shape of a laterally elongated clepsydra. As the solvent evaporates, the liquid retains its shape and, decreasing in volume, confines more and more the sub-micron spheres into a colloidal crystal in the shape of a double triangular prism (Figure 6e). Second, the extremely thin neck of the clepsydra can have the thickness of a single sphere so that when the fabric is removed, even if the formed crystal adheres to the fabric, this neck becomes the ideal cleavage line. Thus, the remaining part on the substrate keeps its integrity (Figure 6e). This hypothesis is suggested by SEM images in which we can see a very similar prismatic colloidal crystal (Figure 6g) on some fiber surfaces very similar to that formed on the substrate (Figure 6f). These observations could give us some ideas about the complexity of the self-assembly phenomenon, the complexity of which we could use to make shaped colloidal crystals.

The current methods of colloidal-crystal synthesis [30,35,36,37,38] work only with a single solid/liquid/gas interface (Figure 6h) and offer only flat or convex crystals. Triangular prisms are often produced when the “capillary-bridge-mediated-assembly” phenomenon/technique is used [57,58,59]. It is worth noting that in this architecture, two solid/liquid/gas interfaces are involved (Figure 6i). They induce the formation of a horizontal liquid bridge parallel with the substrate. By using a fabric sheet, we also have two solid/liquid/gas interfaces (Figure 6j). However, this time, the liquid bridge is in a vertical position, normal to the substrate. We can see that the liquid bridges between two (and why not more than two?) solids can offer complex shapes where capillary forces can lead to the proper self-assembly of colloids in complex three-dimensional shaped colloidal crystals.

## 4. Conclusions

Patterned colloidal crystals (PCCs) on smooth, flat, and few cm^2^ wide hydrophilic substrates can be obtained by using a polyester fabric for guiding the self-assembly of colloidal sub-micron polystyrene (or silica) and even much larger (20 μm) PS spheres. Curved-substrate (such as cylindrical ones) deposition of PCCs was also achieved, especially when using an elastic fabric. This robust method for PCC synthesis is inexpensive and less sensitive to external parameters.

The unit cells of the patterned colloidal crystal have a concave shape, consisting of prisms (or elongated structures as negative-replica of the fabric fibers at higher sphere concentration and even flatted, donut-shaped structures when folded fabrics were employed) at their borders and deposits corresponding to the fabric knots at their intersections, whereas mainly monolayers of sub-micron spheres close-packed in a hexagonal array occupied most of their interior zones.

The mechanisms responsible for the formation of the border structures of the PCC unit cells imply the concentration of spheres in three-dimensional vertically liquid bridges formed between polyester fibers and hydrophilic substrate and their subsequent compacting during liquid evaporation, followed by tissue detachment from the upper part of the deposit after drying.

Patterned colloidal crystals present interesting optical properties that can be visually observed, such as negative refraction and the super-prism effect with an angular dispersion of ~5 nm/degree, an effect also observed for similar PCCs deposited onto cylindrical surfaces. We used the PCCs prisms as lithographic masks and obtained hierarchically assemblies of gold nanodots after sputtering and calcination. Furthermore, they were employed as templates for shaped inverted opals (IO) synthesized from PS sphere PCCs infiltrated with sodium silicate solutions, dried, and calcined, which preserved the main components if the initial opals (prisms, knots, and inner zones).

## Figures and Tables

**Table 1:** Quantification of the order quality of self-assembly of sub-micron spheres.

	RO [mm^2^]			
Structure	PS (0.15 μm)	PS (0.30 μm)	PS (0.488 μm)	SiO_2_ (0.245 μm)
Prisms	13.0	3.0	2.0	6.0
Knots	~0	~0	~0	~0
The interior of the unit cell	6.0	2.0	1.0	3.0
